# Comparative Analysis of Early COVID‐19 Treatment Efficacy in a Multicentric Regional Cohort in Italy: Emulation of a Series of Target Trials

**DOI:** 10.1002/jmv.70379

**Published:** 2025-05-06

**Authors:** Valentina Mazzotta, Alessandro Cozzi Lepri, Cosmo Del Borgo, Simone Lanini, Silvia Meschi, Silvia Garattini, Silvia Rosati, Valentina Siciliano, Alessandra Vergori, Luigi Coppola, Antonio Falletta, Anna Carraro, Giulia Gramigna, Alessandra Oliva, Elena Matteini, Andrea Gasperin, Giuseppina Giannico, Ilaria Mastrorosa, Giulia Matusali, Alessandra D'Abramo, Raffaella Marocco, Eugenia Milozzi, Carlotta Cerva, Francesca Gavaruzzi, Martina Rueca, Claudia Cimaglia, Pierluca Piselli, Massimo Fantoni, Enrico Girardi, Loredana Sarmati, Claudio M. Mastroianni, Massimo Andreoni, Carlo Torti, Emanuele Nicastri, Fabrizio Maggi, Miriam Lichtner, Andrea Antinori, The Early Treatment for COVID‐19 Lazio Study Group

**Affiliations:** ^1^ Clinical Infectious Diseases Department National Institute for Infectious Diseases Lazzaro Spallanzani IRCCS Rome Italy; ^2^ Centre for Clinical Research, Epidemiology, Modelling and Evaluation (CREME), Institute for Global Health, UCL London UK; ^3^ Infectious Diseases Unit, Santa Maria Goretti Hospital Sapienza University of Rome Latina Italy; ^4^ Clinic Infectious Diseases University of Udine Udine Italy; ^5^ Laboratory of Virology, National Institute for Infectious Diseases Lazzaro Spallanzani IRCCS Rome Italy; ^6^ Dipartimento di Sicurezza e Bioetica Università Cattolica del Sacro Cuore Rome Italy; ^7^ PhD Course in Microbiology, Immunology, Infectious Diseases, and Transplants (MIMIT) University of Rome Tor Vergata Rome Italy; ^8^ Clinic of Infectious Diseases, Tor Vergata University Hospital Rome Italy; ^9^ Department of Public Health and Infectious Diseases Sapienza University of Rome Rome Italy; ^10^ Clinical Epidemiology, National Institute for Infectious Diseases Lazzaro Spallanzani IRCCS Rome Italy; ^11^ Dipartimento di Scienze Mediche e Chirurgiche IRCCS Fondazione Policlinico “A. Gemelli” Roma Rome Italy; ^12^ Scientific Direction, National Institute for Infectious Diseases Lazzaro Spallanzani IRCCS Rome Italy

**Keywords:** antiviral agents, epidemiology, SARS coronavirus, virus classification

## Abstract

Studies comparing all available strategies for the early treatment of mild‐to‐moderate COVID‐19 during the Omicron era are lacking. We included people with mild‐to‐moderate COVID‐19 and at high risk of progressing to severe disease attending five outpatient clinics in Italy over 2022–2023. The primary outcome was the proportion of participants who experienced Day‐30 hospitalization due to COVID‐19 or death. Participants received either nirmatrelvir/ritonavir (NMV/r), molnupiravir (MLP), remdesivir (RDV), sotrovimab (SOT), or tixagevimab/cilgavimab (TIX/CIL). We included 10 038 individuals: females 5052 (50%), median age 71 years (IQR 59–81). In total, 1919 (19%) received SOT, 3732 (37.2%) MLP, 1444 (14%) RDV, 2510 (25%) NMV/r, and 433 (4%) TIX/CIL. Only 1689 (17%) had incomplete vaccination, and 2435 (24.3%) were not immunocompetent. The rate of hospitalization/death was 2.40% (95% CI 2.10–2.71). Unadjusted rates were 0.88% (95% CI 0.55–1.32) for NMV/r, 1.69% (95% CI 1.30–2.15) for MLP, 3.0% (95% CI 1.61–5.08) for TIX/CIL, 3.54% (95% CI 2.76–4.47) for SOT and 5.12% (95% CI 4.05–6.39) for RDV. Weighted analysis showed that NMV/r and MLP were superior to all other interventions. In our population of individuals at high risk of progression to severe disease, there was clinical benefit in using NMV/r or MLP instead of mAbs‐based therapies or RDV.

## 1 Introduction

Early treatment of COVID‐19 represented a high‐priority approach for preventing severe outcomes in high‐risk not‐hospitalized patients. Several antiviral drugs (AvD) and monoclonal antibodies (mAbs) have been authorized for use as early therapy based on data from Phase 3 randomized clinical trials (RCTs) conducted in high‐risk unvaccinated people and when predominantly alpha or delta variants were circulating. According to the results of these placebo‐controlled RCTs, AvD such as nirmatrelvir/ritonavir (NMV/r), remdesivir (RDV), and molnupiravir (MLP) provided 88% [1], 87% [2], and 30% [3], risk reduction for hospitalization or death, by Day 29 from randomization, while second‐generation mAbs sotrovimab (SOT) and tixagevimab/cilgavimab (TIX/CIL) an 85% [4] and 50% [5], respectively.

The postulated lower pathogenicity of the Omicron sublineages [6], together with the increase in the SARS‐CoV‐2 vaccine coverage, dramatically reduced the hospitalization/death rate related to COVID‐19, making it difficult to carry out RCTs to assess if AvD or mAbs were still effective in preventing progression to severe COVID‐19 in high‐risk people, and help answering the question of which drug is more effective in the early treatment. In addition to randomized studies, several analyses of observational real‐world (RW) data have also been carried out. Most of these RW analyses involved comparisons between NMV/r, MLP, or SOT [7, 8, 9, 10] and untreated patients, as well as some comparisons between these specific drugs, while there is little data comparing the effectiveness of RDV or TIX/CIL [11]. These analyses consistently showed that NMV/r and MLP could reduce the risk of hospitalization and death compared to untreated populations during the Omicron era [12, 13, 14, 15, 16], also among older people [17]. At the same time, a large pragmatic RCT conducted in vaccinated people [18] showed no evidence for a difference in failure rate comparing MLP plus usual care versus usual care alone, leading the European Medicine Agency (EMA) to withdraw the marketing authorization for MLP [19] in June 2023. Nevertheless, there are concerns that the participants included were not at sufficiently high risk of severe disease. Therefore, there is a need for other studies including a more relevant target population [20]. Furthermore, the advent of the Omicron sublineages has questioned the efficacy of mAbs because of their reported decrease in the in vitro neutralization activity against the newly circulating subvariants [21, 22, 23, 24]. This led, in January 2023, to the U.S. Food and Drug Administration (FDA) no longer authorizing the clinical use of mAbs and the National Institutes of Health (NIH) COVID‐19 panel recommending only AvD for the early treatment of COVID‐19 [25]. Conversely, in October of the same year, EMA still advised against stopping the use of mAbs for early treatment [26]. Finally, the National Institute for Health and Care Excellence (NICE) in the UK, accounting for both effectiveness and cost data, recommended NMV/r as the first choice for early treatment of nonhospitalized COVID‐19 patients but still considered SOT, MLP, and RDV as alternative options [27]. In this scenario, there is a need for more solid estimates of the incidence of hospitalization and death in truly high‐risk patients treated with currently recommended therapies for early COVID‐19 in the Omicron era.

## 2 Methods

All adults who accessed outpatient facilities in five infectious diseases centers in the Lazio Region (as part of an AIFA program started in March 2021) with a confirmed diagnosis of SARS‐CoV‐2 infection by an antigenic or molecular nasopharyngeal swab (NPS) and mild or moderate symptoms of COVID‐19 for 7 days or less and classified to be at high risk for progression to severe COVID‐19 were eligible for treatment and included in this observational study. For the exact definition of mild‐moderate symptoms and high‐risk for progression used we refer to WHO scores [28], NIH COVID‐19 treatment guidelines [25], and AIFA criteria (Supporting Information S2: Target Populations).

All patients were treated between January 2022 and May 2023 (the Omicron era) with RDV, MLP, NMV/r, SOT, and TIX/CIL. The choice of the drug used was based on the physician's assessment and judgment of the time from the onset of symptoms (according to different fact sheets), the availability of drugs (which has changed over time), and the presence of contraindications to specific drugs (according to different fact sheets).

All included individuals have signed a written informed consent to participate in the study. The observational multicentric study protocol and the informed consent have been approved by the Ethical Committee of the National Institute for Infectious Diseases Lazzaro Spallanzani (Approval Number: n. 380, 09/30/2021. FAV del Registro delle Sperimentazioni 2020/2021). On the day of the first evaluation (baseline), demographic and clinical data were collected, including information on time from symptoms onset, vaccination status, and comorbidities. Treatment was started on the same day of the evaluation according to NIH guidelines [25]. Remdesivir was given as a 3‐day course in three consecutive outpatient visits. Only individuals who completed the full treatment course have been included here. The SARS‐CoV‐2 variant was established either directly, by sequencing where available, or indirectly, inferred from the calendar period of baseline date based on Italian regional surveillance data. The primary outcome was the proportion of participants who experienced COVID‐19‐related clinical failure, defined as hospitalization due to the development of severe COVID‐19 or death from any cause over Days 0–30. Follow‐up information was collected by in‐presence or by telephonic visit at a single time point at Day 30 for those who did not return to hospital after treatment. We did not analyze safety data in this analysis as the collection was incomplete and not standardized in all participating sites.

The main characteristics of the study population assessed at baseline were compared by treatment strategies using *χ* ^2^ or Kruskal–Wallis test, as appropriate. Unadjusted risks of hospitalization by Day 30 were calculated with 95% CI for each of the considered treatments, then we used a weighted pooled logistic regression model to approximate the parameters of a marginal structural Cox model [28, 29, 30] to estimate the relative hazards of hospitalization/death by Day 30 in separate 2‐arms parallel emulated trials, encompassing all possible 2‐arm comparisons between the drugs (a total of 10 possible pairwise comparisons). The main inclusion criteria were applicable for all the 2‐arm comparisons except for severe hepatic and renal impairment. Moreover, for the trial emulation we have used additional specific inclusion criteria accounting for the observed duration of symptoms and the exact month of accessing the outpatient setting (Supporting Information S2: Trial Specification Table). In a sensitivity analysis, we further included participating site in the propensity score model for treatment and censoring weights. For details regarding the statistical analysis, we refer to the Supporting Information S3: Technical Documentation.

To further control for possible violations of the positivity assumptions (as both hepatic and renal impairment may have dramatically lowered the probability of using specific drugs) as well as to detect a signal that some drugs may have performed differently in subgroups of participants with specific profiles, we also conducted post hoc comparisons in subsets of the study population (using stratified marginal models and results shown as forest plots) and reported the *p* values for interaction. All participants had complete data for all the variables included for analysis. All analyses have been performed using SAS V9.4 (SAS Institute Inc. 2023. *SAS/STAT 15.3 User's Guide*. Cary, NC: SAS Institute Inc.) and Stata v18 (StataCorp. 2023. *Stata Statistical Software: Release 18*. College Station, TX: StataCorp LLC).

## 3 Results

Among the 10 038 participants treated between January 2022 and May 2023, 1919 (19.1%) received SOT, 3732 (37.2%) MLP, 1444 (14.4%) RDV, 2510 (25.0%) NMV/r and 433 (4.3%) TIX/CIL. The main characteristics of participants according to the treatment that they received are reported in Table 1. Median age was 71 years (IQR 59–81) and 5052 (50.3%) were females. Participants with none or incomplete vaccination were 1689 (16.8%), and immunocompromised status was established for 2435 (24.3%) subjects. Due to treatment contraindications, there were some imbalances in the prevalence of baseline risk factors for disease progression by treatment arms, for example, participants with renal impairment were treated mostly with SOT or MLP, while those with hepatic impairment preferentially with mAbs (Table 1). Sequencing data were available for 1742 participants (17.3%), and the majority (37%) were infected with Omicron BA4.5.

**Table 1:** Main characteristics at enrollment by intervention.

		Intervention started						
		SOT	MLP	RDV	NMV/r	TIX/CIL		Total
Characteristics		*N* = 1919	*N* = 3732	*N* = 1444	*N* = 2510	*N* = 433	*p* ^e^	*N* = 10 038
Gender, *n* (%)							< 0.001	
Female		988 (51.5%)	1815 (48.6%)	676 (46.8%)	1353 (53.9%)	220 (50.8%)		5052 (50.3%)
Age, years							< 0.001	
Median (IQR)		68 (55, 79)	76 (66, 84)	72 (60, 81)	65 (52, 74)	68 (56, 78)		71 (59, 81)
Older than 65, *n* (%)		1058 (55.1%)	2846 (76.3%)	952 (65.9%)	1213 (48.3%)	242 (55.9%)	< 0.001	6311 (62.9%)
Days from symptoms onset to treatment							< 0.001	
Median (IQR)		3 (2, 5)	3 (2, 4)	3 (2, 5)	3 (2, 4)	3 (2, 4)		3 (2, 4)
Comorbidities/risk factors, *n* (%)								
Diabetes		293 (15.3%)	800 (21.4%)	269 (18.6%)	338 (13.5%)	60 (13.9%)	< 0.001	1760 (17.5%)
Obesity (BMI > 30)		218 (11.4%)	594 (15.9%)	204 (14.1%)	362 (14.4%)	60 (13.9%)	< 0.001	1438 (14.3%)
CVD		997 (52.0%)	2802 (75.1%)	984 (68.1%)	1273 (50.7%)	224 (51.7%)	< 0.001	6280 (62.6%)
COPD		357 (18.6%)	860 (23.0%)	334 (23.1%)	463 (18.4%)	92 (21.2%)	< 0.001	2106 (21.0%)
Renal impairment		453 (23.6%)	342 (9.2%)	72 (5.0%)	150 (6.0%)	193 (44.6%)	0.001	1210 (12.1%)
Hepatic disease		61 (3.2%)	65 (1.7%)	35 (2.4%)	39 (1.6%)	10 (2.3%)	< 0.001	210 (2.1%)
Cancer		326 (17.0%)	187 (5.0%)	94 (6.5%)	208 (8.3%)	64 (14.8%)	< 0.001	879 (8.8%)
Immunocompromised status^f^		727 (37.9%)	566 (15.2%)	342 (23.7%)	667 (26.6%)	133 (30.7%)	< 0.001	2435 (24.3%)
Neurologic disease		78 (4.1%)	53 (1.4%)	18 (1.2%)	39 (1.6%)	14 (3.2%)	< 0.001	202 (2.0%)
Vital signs at baseline								
Fever (> 37.5°C), *n* (%)		26 (2.4%)	11 (1.1%)	4 (1.1%)	8 (1.0%)	4 (1.4%)	0.079	53 (1.5%)
BMI, median (IQR)		25.33 (22.66, 28.89)	26.23 (23.84, 29.41)	25.71 (23.44, 29.32)	25.71 (23.04, 29.33)	24.91 (22.86, 28.72)	< 0.001	25.86 (23.34, 29.38)
Vaccination status, *n* (%)							< 0.001	
None or incomplete^a^		232 (12.1%)	565 (15.1%)	417 (28.9%)	410 (16.3%)	65 (15.0%)		1689 (16.8%)
Full not recent^b^		1056 (55.0%)	2054 (55.0%)	639 (44.3%)	1583 (63.1%)	290 (67.0%)		5622 (56.0%)
Full recent^c^		631 (32.9%)	1113 (29.8%)	388 (26.9%)	517 (20.6%)	78 (18.0%)		2727 (27.2%)
Calendar period according to the main circulating VoC, *n* (%)							< 0.001	
January–March 2022 (BA.1)	815 (42.5%)		1153 (30.9%)	472 (32.7%)	320 (12.7%)	40 (9.2%)	< 0.001	2800 (27.9%)
April–June 2022 (BA.2)	468 (24.4%)		1059 (28.4%)	444 (30.7%)	931 (37.1%)	58 (13.4%)	< 0.001	2960 (29.5%)
July–December 2022 (BA.4/5)	486 (25.3%)		1312 (35.2%)	507 (35.1%)	1085 (43.2%)	291 (67.2%)	< 0.001	3681 (36.7%)
January–February 2023 (BQ.1)	58 (3.0%)		187 (5.0%)	11 (0.8%)	95 (3.8%)	43 (9.9%)	< 0.001	394 (3.9%)
March–May 2023 (XBB)	92 (4.8%)		21 (0.6%)	10 (0.7%)	79 (3.1%)	1 (0.2%)	< 0.001	203 (2.0%)

Abbreviations: BMI, body mass index; CVD, cardiovascular disease; COPD, chronic obstructive pulmonary disease; IQR, interquartile range.

^a^ Incomplete: < 3 doses.

^b^ Full: > 3 doses.

^c^ Not recent: the last dose administered more than 120 days before.

^d^ Recent: the last dose administered < 20 days before.

^e^ *χ* ^2^ or Kruskal–Wallis test as appropriate.

^f^Defined as primary or secondary immunodeficiency and/or treatment with immunosuppressive agents.

**Figure 1:**
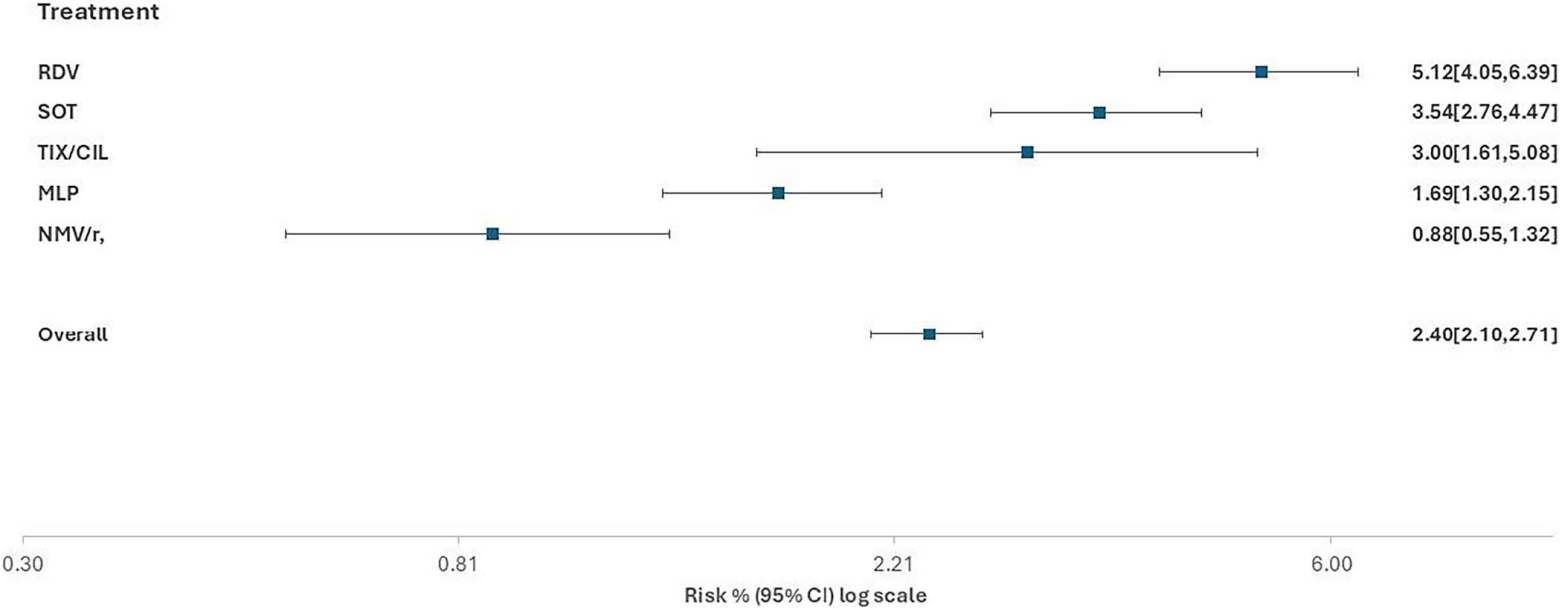
Crude incidence rates (natural course).

Overall, 240 events (COVID‐19‐related hospitalization/death) were observed in the treated population observed in the Omicron era, with an overall rate of hospitalization or death of 2.40% (95% CI 2.10–2.71). Crude cumulative incidence of clinical failure according to treatment (as by natural course) was 0.88% (95% CI 0.55–1.32) for NMV/r, 1.69% (95% CI 1.30–2.15) for MLP, 3.0% (95% CI 1.61–5.08) for TIX/CIL, 3.54% (95% CI 2.76–4.47) for SOT, and 5.12% (95% CI 4.05–6.39) for RDV (Figure 1).

In the unweighted analysis, NMV/r was superior to all other interventions (*p* < 0.004). MLP was superior to SOT (*p* < 0.001), RDV (*p* < 0.001), and TIX/CIL (*p* = 0.07), although the result was borderline nonsignificant for this latter contrast. In turn, there was some evidence that SOT was superior to RDV (*p* = 0.02), but there was no evidence for a difference when compared to TIX/CIL (*p* = 0.82, Table 2). Interestingly, after controlling for confounding pathways, all these results were confirmed except for the contrasts RDV versus SOT (wRH = 1.35, 95% CI: 0.93, 1.95, *p* = 0.11) and NMV/r versus MLP (wRH = 0.82, 95% CI: 0.48–1.42, *p* = 0.48, Table 2). Results were similar when we used the risk difference scale instead of the RH (Supporting Information S1: Table 1) and after further controlling for participating sites in the propensity score model (Supporting Information S1: Table 2).

**Table 2:** Unweighted and weighted hazard ratios of hospitalization or death—all pairwise contrasts.

	Marginal hazard ratios (HR) of hospitalization^a^ or death by Day 30			
	Unweighted HR (95% CI)	*p*	Weighted^b^ HR (95% CI)	*p*
Contrasts with NMV/r as intervention				
NMV/r vs. MLP	0.48 (0.29, 0.79)	0.004	0.82 (0.48, 1.42)	0.482
NMV/r vs. RDV	0.16 (0.10, 0.26)	< 0.001	0.23 (0.14, 0.39)	< 0.001
NMV/r vs. SOT	0.23 (0.14, 0.39)	< 0.001	0.31 (0.17, 0.54)	< 0.001
NMV/r vs. TIX/CIL	0.22 (0.08, 0.62)	0.004	0.38 (0.12, 1.23)	0.105
Contrasts with MLP as intervention				
MLP vs. RDV	0.32 (0.22, 0.45)	< 0.001	0.29 (0.20, 0.41)	< 0.001
MLP vs. SOT	0.46 (0.33, 0.66)	< 0.001	0.49 (0.33, 0.74)	< 0.001
MLP vs. TIX/CIL	0.47 (0.21, 1.05)	0.065	0.46 (0.18, 1.18)	0.106
Contrast with RDV as intervention				
RDV vs. SOT	1.46 (1.05, 2.03)	0.024	1.35 (0.93, 1.95)	0.110
RDV vs. TIX/CIL	0.81 (0.32, 2.06)	0.664	0.44 (0.16, 1.24)	0.121
Contrasts with SOT as intervention				
SOT vs. TIX/CIL	1.10 (0.49, 2.44)	0.822	1.13 (0.50, 2.57)	0.769

a Due to COVID‐19.

^b^ Weighted for age, immunocompromised status, vaccination status, time from symptoms onset, moderate hepatic and renal impairment, calendar time, and censoring using IPW.

**Figure 2:**
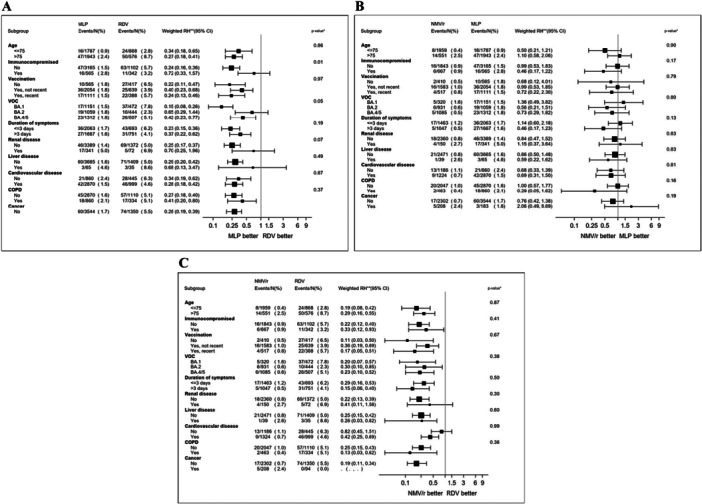
Forest plot of the comparison between antiviral drugs in subsets. (A) Molnupiravir versus remdesivir (MLP vs. RDV). (B) Nirmatrelvir/ritonavir versus molnupiravir (NMV/r vs. MLP). (C) Nirmatrelvir/ritonavir versus remdesivir (NMV/r vs. RDV). *Note: p* value corresponds to the test for interaction between treatment and stratification factor; **RH from fitting a marginal structural model.

Overall, there was little evidence that important effect measure modifiers for the difference between interventions existed. The only significant interaction was observed for immunosuppression and the contrast MLP versus RDV (*p* = 0.01). Specifically, while MLP was still superior to RDV in participants without immunosuppression, this difference was largely attenuated in the group with immunodeficiency (Figure 2A). Regarding the other contrasts, we found no evidence for significant interactions for the comparison NMV/r versus MLP (Figure 2B) and NMV/r versus RDV (Figure 2C). In other words, NMV/r was superior to RDV in all subgroups, and the contrast between NMV/r versus MLP remained inconclusive regardless of the subsets analyzed (Supporting Information S1: Supporting Material (Figures 1–7)).

## 4 Discussion

The question of which is the best option for the early treatment of COVID‐19 in high‐risk people remains largely unresolved. In the absence of results from RCTs, in vitro experiments data and RW studies have been mainly used to shape clinical guidelines and have led to inconsistent recommendations across regulatory boards [31, 32]. Also, evidence is fragmented as none of these previous studies were able to compare all the main treatment options (both AvD and mAbs) available for the early treatment of COVID‐19 in high‐risk populations. In addition, to our knowledge, the present analysis is the first to include a target population of 80% vaccinated individuals with > 20% with immunodeficiency disorders.

The key finding of our analysis is that oral antivirals (NMV/r and MLP) appear to have superior clinical efficacy compared to the two mAbs (SOT or TIX/CIL) or the other antiviral RDV. Our findings for the contrast of NMV/r over SOT are inconsistent with those of previous reports. Specifically, two large population studies of nonhospitalized high‐risk persons with COVID‐19 collected in primary care in England [8] and electronic health record (EHR) data in Wales [33], performed, respectively, when the Omicron subvariants BA.2, BA.5, or BQ.1 or BA.1/BA.2 were dominant, showed weak evidence for a difference in the risk of severe COVID‐19 outcomes between people receiving NMV/r or SOT. There could be multiple reasons for this discrepancy. First, the case mix of the target populations as our population was enriched with individuals at higher risk of disease progression than the average person attending primary care facilities or included in EHR. In addition, both these UK analyses employed standard regression models adjusted for covariates (using individual features or propensity scores adjustment) and not a marginal model as in our approach. From a virological point of view, it is not surprising that antivirals which act directly against the virus protease may retain greater efficacy than mAbs which are tailored to specific viral variants.

We have also shown superior clinical efficacy of NMV/r over TIX/CIL (> 3‐fold) and RDV (> 5‐fold reduction in risk of hospitalization/death). The first result is not unexpected and entirely consistent with those shown by a recent pooled analysis of RCTs, which, however, did not compare NMV/r with RDV and lacked statistical power for the contrast NMV/r versus SOT [34, 35]. In addition, NMV/r appeared to be superior to RDV also in an observational study comparing antiviral potency. This was shown both in a direct comparison [36] and from indirect evidence comparing two separate placebo‐controlled trials; indeed, when compared against placebo, a higher antiviral activity could be shown for NMV/r [1] but not for RDV [2].

Interestingly, the most controversial contrast regards the comparison between the two oral direct antiviral agents, NMV/r and MLP. In studies using a virological endpoint, NMV/r was shown to have higher antiviral activity than MLP both in RCTs [37] and the observational setting [36]. However, these results are difficult to interpret as there is currently no consensus regarding whether virological response is a valid surrogate marker for clinical progression. Two previous large studies showed that NMV/r was more effective than MLP in reducing the risk of all‐cause mortality in individuals with COVID‐19 [9, 38]. Again, there were also some methodological differences compared to our analysis as in the first study by Wan et al. [38], both nonhospitalized and hospitalized individuals were included (although the results were similar in the two populations), and standard propensity score matching analysis was used. The second study [9] used data collected from an Italian‐wide drug registry and used a different construction of the propensity score model to determine the weights (machine learning vs logistic regression with linear predictor in our analysis). Despite these differences, our results were consistent with those shown by Torti and colleagues showing a 16% reduction in risk for NMV/r with the 95% CI largely overlapping with ours.

In two recent placebo‐controlled RCTs conducted in the Omicron era, the failure rate was around 0.80% for both people treated NMV/r [39] and MLP [18], without evidence for a clinical benefit when using AvD instead of placebo, questioning the usefulness of these treatments. However, these recent trials comprised mainly individuals with an average lower risk of progression compared to ours, so the results are not directly comparable. Reported high risk of severe and persistent disease in specific high‐risk groups also during the Omicron era, despite full vaccination, suggests that the use of AvD should still be recommended for these fragile populations [40, 41, 42].

Regarding the comparison between MLP and RDV, as mentioned above, overall, our data carry strong evidence for clinical benefit in using MLP instead of RDV. However, this difference was largely attenuated in people with severe immunodeficiency. The reasons for this interaction are unclear, but the aim of this post hoc analysis was merely to evaluate whether the main comparisons were consistent across subgroups and to guide future studies. Finally, our analysis does not carry evidence for a difference in risk of progression to severe COVID‐19 between the two mAbs (SOT and TIX/CIL) or between the two mAbs and RDV, as reported by previous sporadic data available on these comparisons [11, 37] suggesting that all these are equivalently inferior to NMV/r and MLP.

Some limitations need to be mentioned. First, our results are valid under the usual assumptions in causal inference (e.g., no unmeasured confounding, positivity, consistency, models were correctly specified, etc.). The distribution of stabilized weights, propensity score density, and standardized mean differences plots did not indicate violations of positivity or model misspecifications. Data on IgG SARS‐CoV‐2 serology were available only for participants enrolled in one of the five centers. Although inclusion criteria have been tailored to individual trials, because of the granularity of the information on some drug contraindications (i.e., immunosuppressive therapy after solid organ transplantation and use of some anticoagulants for NMV/r), we cannot rule out that a small proportion of participants were not eligible for specific comparisons. However, results were similar in subset analyses, including only immunocompetent participants or those free from cardiovascular disease. Being a collaborative effort of existing clinical studies, data have not been collected according to a standardized operating procedure, so despite harmonization, we cannot rule out residual confounding due to imprecise measurement of some of the covariates. Because we included individuals at high risk of progression to severe disease, it was unethical to leave them without treatment, and therefore, we were unable to include a group of untreated individuals to estimate the underlying risk of hospitalization/death under natural course.

In conclusion, our analysis of large RW population (> 10 000 people) of mostly vaccinated individuals infected with Omicron and at high risk of progression to severe COVID‐19 disease shows a clinical benefit of using NMV/r or MLP instead of mAbs‐based therapies or RDV. The relevance of these data needs to be reconciled with recent data showing no effect of antivirals against placebo, although in healthier populations. In the absence of randomized comparisons, our data represent the highest level of available evidence to guide early treatment decisions in people with high risk of severe COVID‐19 disease according to specific drug contraindications and limitations.

## Author Contributions

Andrea Antinori and Valentina Mazzotta conceptualized and designed the study. Andrea Antinori, Valentina Mazzotta, and Alessandro Cozzi Lepri wrote the first draft of the manuscript and referred to appropriate literature. Alessandro Cozzi Lepri was also the main person responsible for formal data analysis. Cosmo Del Borgo, Silvia Garattini, Silvia Rosati, Valentina Siciliano, Alessandra Vergori, Luigi Coppola, Antonio Falletta, Anna Carraro, Alessandra Oliva, Elena Matteini, Andrea Gasperin, Alessandra D'Abramo, Ilaria Mastrorosa, Raffaella Marocco, Eugenia Milozzi, Carlotta Cerva, and Francesca Gavaruzzi enrolled the patients and performed the follow‐up visits. Silvia Meschi, Giulia Matusali, Martina Rueca, Giulia Gramigna and Fabrizio Maggi performed the viral sequencing. Giuseppina Giannico, Claudia Cimaglia, and Pierluca Piselli were responsible for data curation. Massimo Fantoni, Enrico Girardi, Loredana Sarmati, Claudio M. Mastroianni, Massimo Andreoni, Carlo Torti, Emanuele Nicastri, Fabrizio Maggi, and Miriam Lichtner revised the manuscript content and reviewed and edited it. All authors agreed with and approved the final version of the manuscript.

## Ethics Statement

The observational multicentric study protocol and the informed consent have been approved by the Ethical Committee of the National Institute for Infectious Diseases Lazzaro Spallanzani (Approval Number: n. 380, 09/30/2021. FAV del Registro delle Sperimentazioni 2020/2021).

## Consent

All included individuals have signed a written informed consent to participate in the study.

## Conflicts of Interest

Alessandro Cozzi‐Lepri received research grants or contract from Icona Foundation Study (money paid to UCL) and European Union [Title: “EuCARE: European Cohorts of Patients and Schoolsto Advance Response to Epidemics.” Grant Agreement No. 101046016 (money paid toUCL). Valentina Mazzotta received institutional research grant from Gilead Science, speaking honorariafor congress from ViiV Healthcare e consultation fees for Viatris and Gilead Science. Valentina Siciliano declares payment or honoraria for lectures, presentations, speakers bureaus, manuscript writing or educational events by Pfizer Support for attending meetings by Astrazeneca. Simone Lanini declares consulting fees by GSK, ViiV, MSD and Gilead Support for attending meetings by MSD, Gilead, ViiV. Silvia Meschi declares receipt of equipment, materials, drugs, medical writing, gifts or other services by Abbott srl. Alessandra Vergori declares consulting fees by Gilead Sciences ViiV Healthcare Support for attending meetings by MSD, Gilead, ViiV, Astrazeneca. Enrico Girardi declares Grants or contracts from any entity (if not indicated in item #1 above) by Gilead (paid to INMI) and Mylan (paid to INMI) payment or honoraria for lectures, presentations, speakers bureaus, manuscript writing or educational events by Gilead. Loredana Sarmati declares payment or honoraria for lectures, presentations, speakers bureaus, manuscript writing or educational events by Merck, Gilead, Abbvie, Angelini, Astra Zeneca, GSK Support for attending meetings and/or travel by Gilead, Merck, Pfizer. Emanuele Nicastri declares consulting fees from Gliead Science payment or honoraria for lectures, presentations, speakers bureaus, manuscript writing or educational events by Roche Support for attending meetings and/or travel by Pharmamar Participation on a Data Safety Monitoring Board or Advisory Board by Takeda and Valneva. Andrea Antinori declares Grants or contracts from any entity (if not indicated in item #1 above) by Gilead (paid to INMI) Astra Zeneca (paid to INMI) ViiV Healthcare (paid to INMI) Consulting fees from Gilead, Astra Zeneca, Pfizer, Janssen‐Cilag, Moderna, GSK, Merck, ViiV Healthcare Payment or honoraria for lectures, presentations, speakers bureaus, manuscript writing or educational events by Gilead, Moderna, Pfizer, Merck, ViiV Healthcare support for attending meetings and/or travel by Gilead, Astra Zeneca. The other authors declare no conflicts of interest.

## Supporting information

4 Supplementary material JMV revision.

5 Emulation table JMV final.

6 Technical documentation JMV revision.

## Data Availability

Anonymized participant data will be made available upon reasonable requests directed to the corresponding author. Proposals will be reviewed and approved by investigators and collaborators on the basis of scientific merit. After approval of a proposal, data can be shared through a secure online platform after signing a data access agreement. As a group, we are very open to collaboration and in involving other researchers in our work. However, we strongly feel that we cannot make a full data set publicly available, the main reason being confidentiality.
