# Supplementary material for: Comparative Analysis of Early COVID‐19 Treatment Efficacy in a Multicentric Regional Cohort in Italy: Emulation of a Series of Target Trials
Source: J Med Virol. 2025 May 6;97(5):e70379. doi: 10.1002/jmv.70379 (PMC12054396; doi:10.1002/jmv.70379)
Supplement: Supplementary file 1 — 4 Supplementary material JMV revision. [file JMV-97-e70379-s002.docx]

Supplemental material

**Comparative analysis of early COVID-19 treatment efficacy in a multicentric cohort of an Italian Region: emulation of a target trial**

*Mazzotta V, Cozzi Lepri A, Del Borgo C, et al.*

Supplementary Table 1: Weighted risk differences of hospitalisation or death - all pairwise contrasts.

Supplementary Table 2: Unweighted and weighted hazard ratios of hospitalisation or death - all pairwise contrasts – analysis further controlled for participating site.

Supplementary Figure 1: Forest plot of the comparison between Nirmatrelvir/ritonavir and Sotrovimab (NMV/r vs SOT) in subsets

Supplementary Figure 2: Forest plot of the comparison between Nirmatrelvir/ritonavir and Tixagevimab/cilgavimab (NMV/r vs TIX/CIL) in subsets

Supplementary Figure 3: Forest plot of the comparison between Molnupiravir and Sotrovimab (MLP vs SOT) in subsets

Supplementary Figure 4: Forest plot of the comparison between Molnupiravir and Tixagevimab/cilgavimab (MLP vs TIX/CIL) in subsets

Supplementary Figure 5: Forest plot of the comparison between Remdesivir and Sotrovimab (RDV vs SOT) in subsets

Supplementary Figure 6: Forest plot of the comparison Remdesivir and Tixagevimab/cilgavimab (RDV vs TIX/CIL) in subsets

Supplementary Figure 7: Forest plot of the comparison between Sotrovimab and Tixagevimab/cilgavimab (SOT vs TIX/CIL) in subsets

Supplementary Table 1: Weighted risk differences (RDx1,000) of hospitalisation or death^&^ - all pairwise contrasts.

|  | **Weighted^*^ RD (95% CI)**    -0.09 (-0.31; +0.14)  -1.28 (-1.69; -0.88)  -0.82 (-1.15; -0.05)  -0.46 (-1.00; +0.08)    -1.49 (-2.00; -0.98)  -0.72 (-1.16; -0.28)  -0.45 (-1.20; +0.30)    +0.38 (-0.14;+0.90)  +0.66 (-0.18; +1.50)    +0.20 (-0.52; +0.93) | **p-value**    0.45  <.001  <.001  0.09    <.001  0.001  0.24    0.15  0.12    0.59 |
| --- | --- | --- |
| ***Contrasts with NMV/r as intervention*** |  |  |
| NMV/r vs. MLP |  |  |
| NMV/r vs. RDV |  |  |
| NMV/r vs. SOT |  |  |
| NMV/r vs. TIX/CIL |  |  |
| ***Contrasts with MLP as intervention*** |  |  |
| MLP vs. RDV |  |  |
| MLP vs. SOT |  |  |
| MLP vs. TIX/CIL |  |  |
| ***Contrast with RDV as intervention*** |  |  |
| RDV vs. SOT |  |  |
| RDV vs. TIX/CIL |  |  |
| ***Contrasts with SOT as intervention*** |  |  |
| SOT vs. TIX/CIL |  |  |
|  |  |  |
| *Weighted for age, immunocompromised status, vaccination statis, time from symptom onset, moderate hepatic and renal impairment and calendar time using AIPW | | |
| \| ^&^due to COVID-19 \| \| --- \| | | |

Supplementary Table 2: Unweighted and weighted hazard ratios of hospitalisation or death - all pairwise contrasts – analysis controlled for participating site.

|  | **Marginal hazard ratios (HR) of hospitalisation^&^ or death by day 30** | | | |
| --- | --- | --- | --- | --- |
|  | **Unweighted HR (95% CI)** | **p-value** | **Weighted^*^ HR (95% CI)** | **p-value** |
| ***Contrasts with NMV/r as intervention*** |  |  |  |  |
| NMV/r vs. MLP | 0.48 (0.29, 0.79) | 0.004 | 0.78 (0.45, 1.34) | 0.367 |
| NMV/r vs. RDV | 0.16 (0.10, 0.26) | <.001 | 0.20 (0.12, 0.35) | <.001 |
| NMV/r vs. SOT | 0.23 (0.14, 0.39) | <.001 | 0.32 (0.17, 0.61) | <.001 |
| NMV/r vs. TIX/CIL | 0.22 (0.08, 0.62) | 0.004 | 0.40 (0.11, 1.40) | 0.150 |
| ***Contrasts with MLP as intervention*** |  |  |  |  |
| MLP vs. RDV | 0.32 (0.23, 0.45) | <.001 | 0.28 (0.20, 0.41) | <.001 |
| MLP vs. SOT | 0.46 (0.33, 0.66) | <.001 | 0.44 (0.28, 0.68) | <.001 |
| MLP vs. TIX/CIL | 0.47 (0.21, 1.05) | 0.066 | 0.43 (0.18, 1.04) | 0.061 |
| ***Contrast with RDV as intervention*** |  |  |  |  |
| RDV vs. SOT | 1.46 (1.05, 2.03) | 0.024 | 1.20 (0.78, 1.84) | 0.398 |
| RDV vs. TIX/CIL | 0.81 (0.32, 2.06) | 0.662 | 0.63 (0.18, 2.23) | 0.479 |
| ***Contrasts with SOT as intervention*** |  |  |  |  |
| SOT vs. TIX/CIL | 1.10 (0.49, 2.44) | 0.824 | 1.25 (0.49, 3.20) | 0.642 |
| ^*^Weighted for age, immunosuppression, history of vaccination, duration of symptoms, hepatic and renal disease, calendar time of infusion, participating hospital and censoring using IPW | | | | |
| ^&^due to COVID-19 | | | | |

**Supplementary Figure 1: Forest plot of the comparison NMV/r vs SOT in subsets**


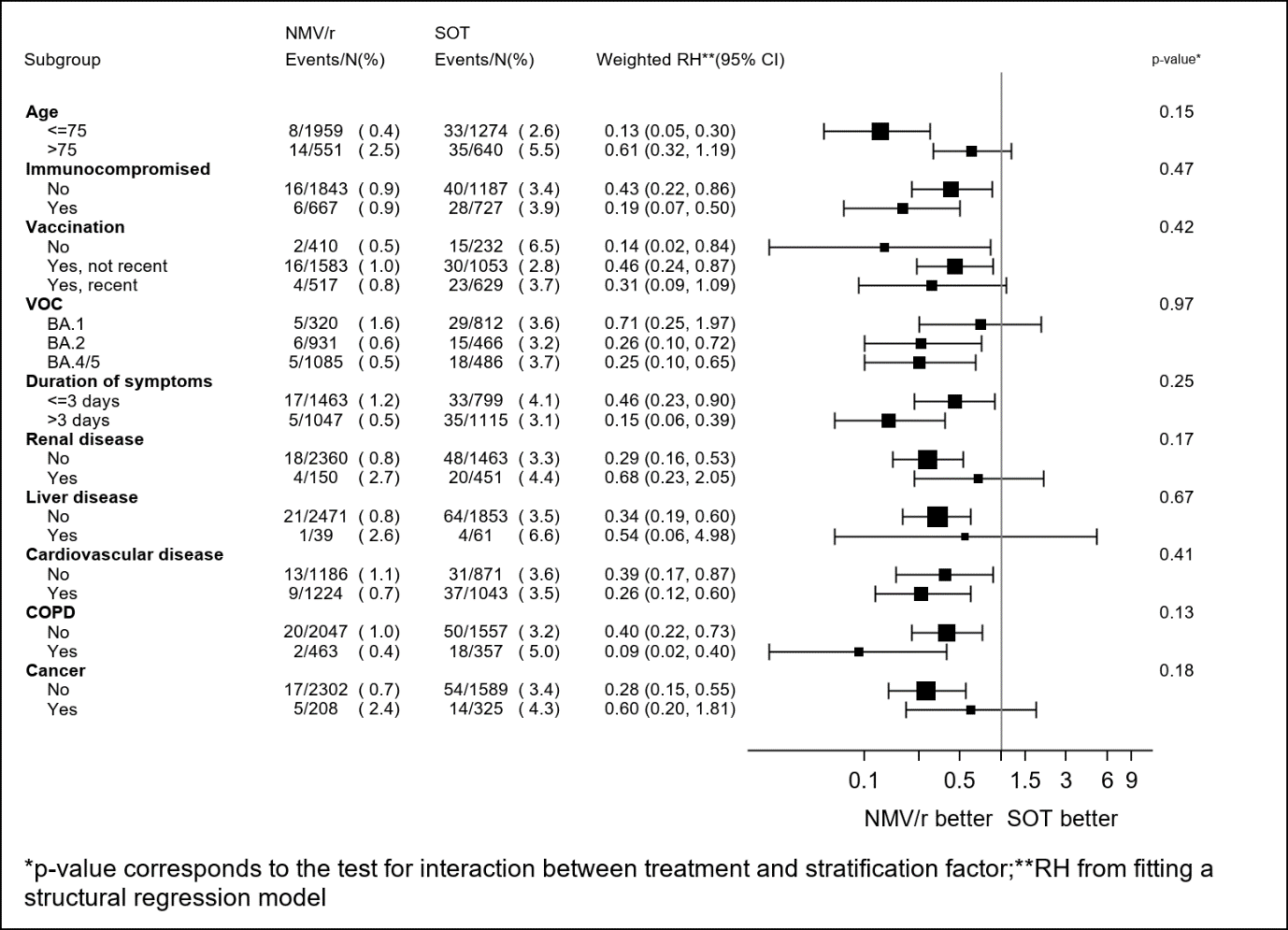


VOC=Variant of concern; COPD=chronic obstructive pulmonary disease

Supplementary Figure 2: Forest plot of the comparison NMV/r vs TIX/CIL in subsets


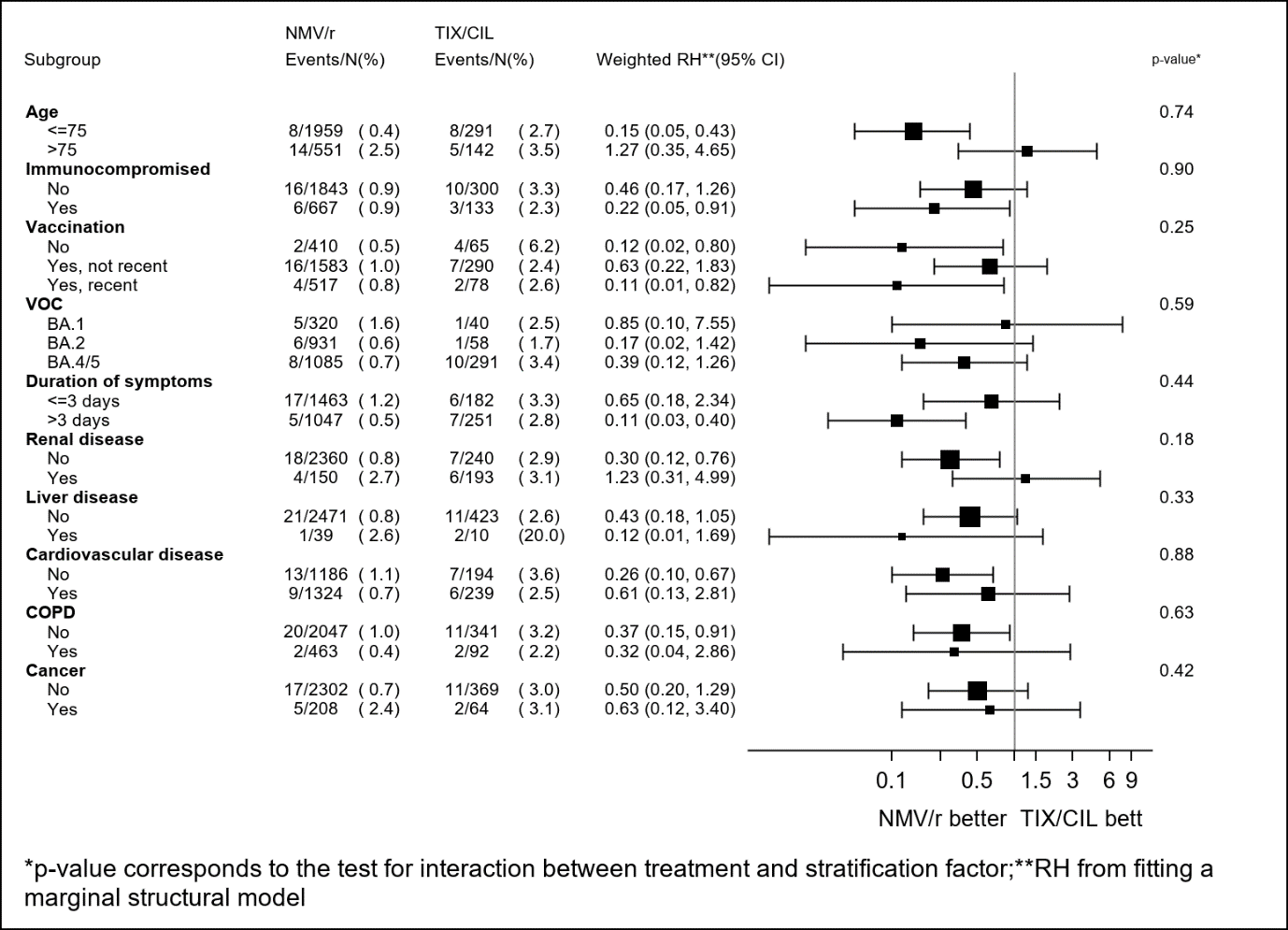


VOC=Variant of concern; COPD=chronic obstructive pulmonary disease

Supplementary Figure 3: Forest plot of the comparison MLP vs SOT in subsets


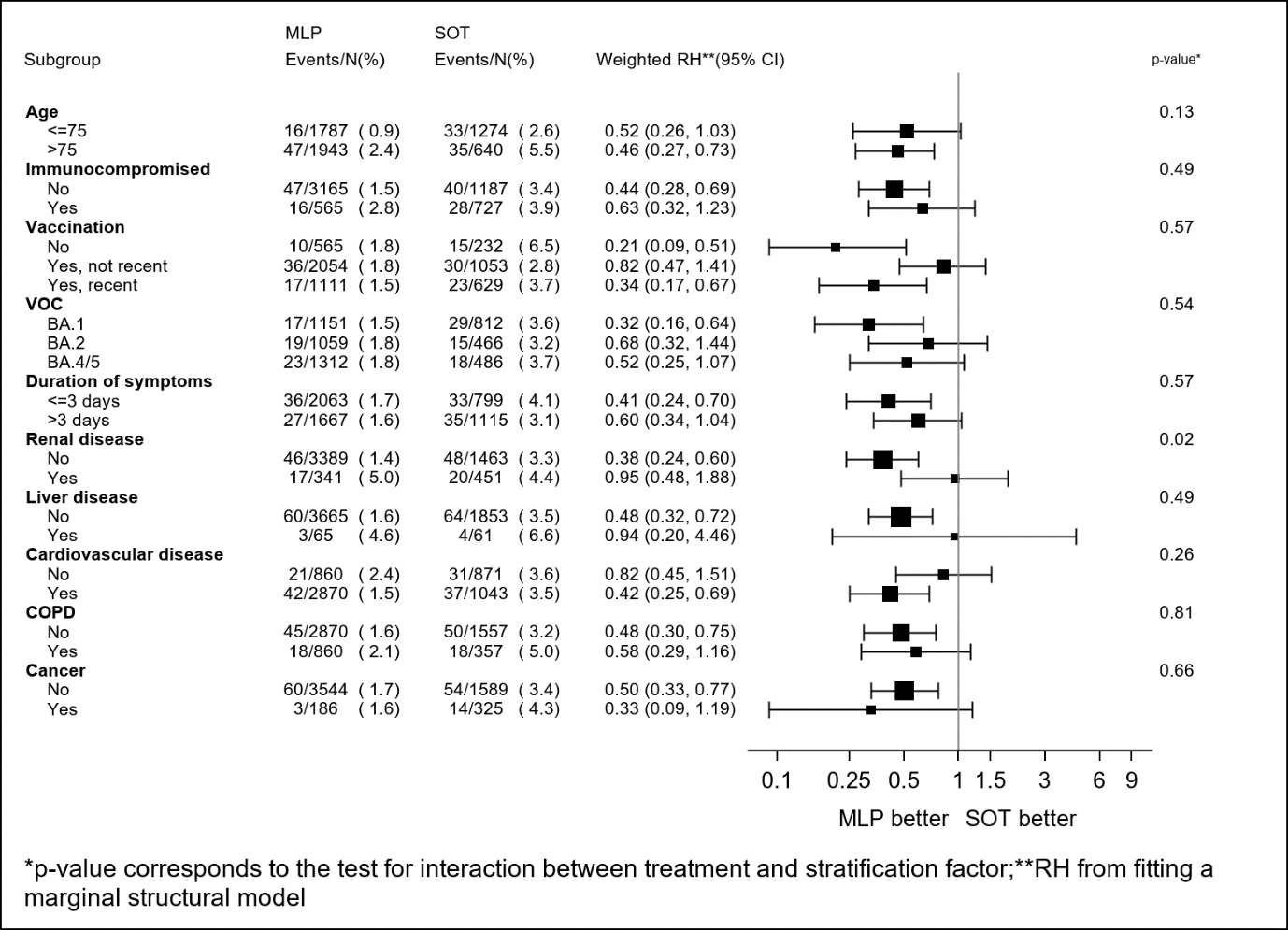


VOC=Variant of concern; COPD=chronic obstructive pulmonary disease

Supplementary Figure 4: Forest plot of the comparison MLP vs TIX/CIL in subsets


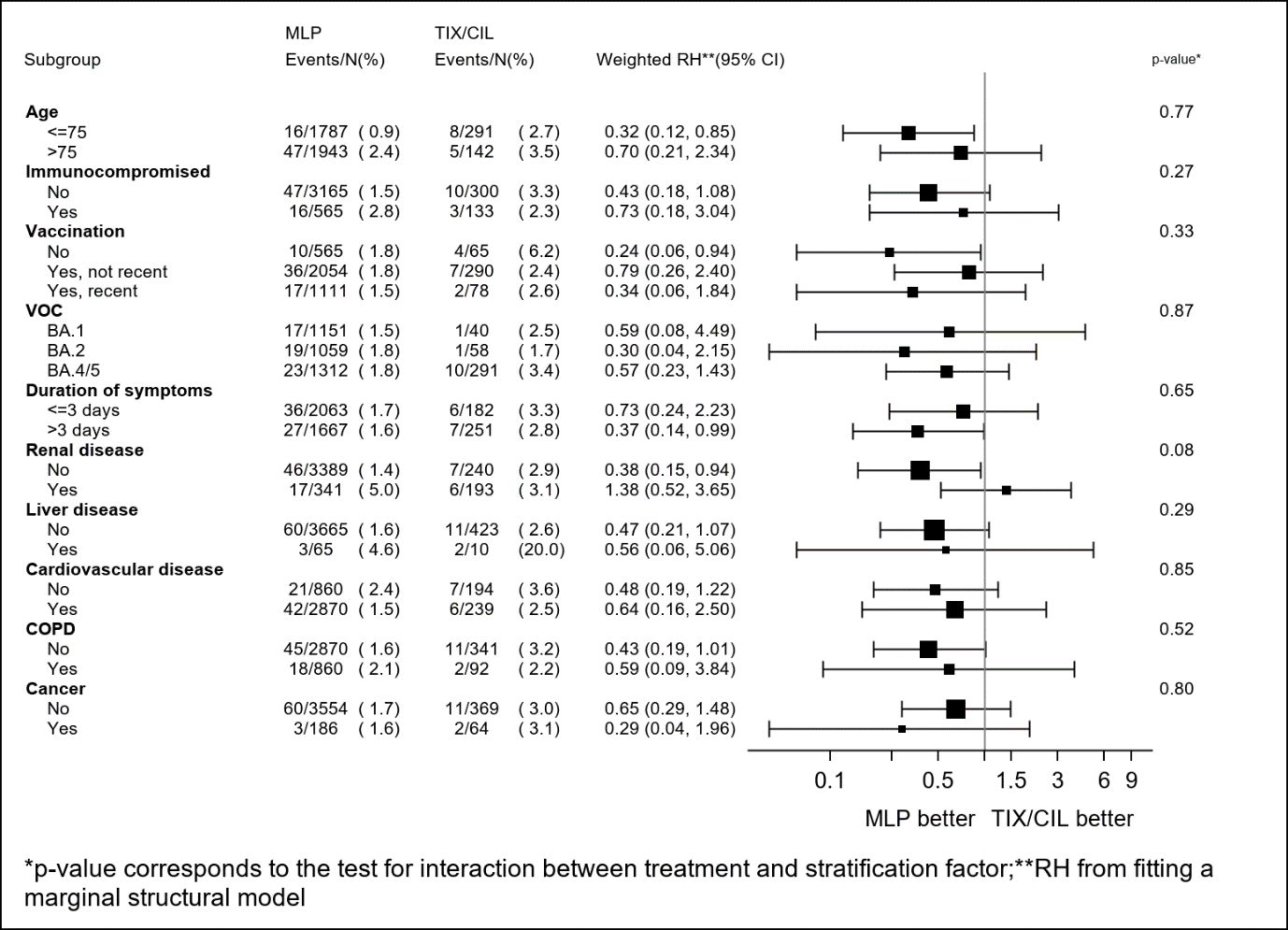


VOC=Variant of concern; COPD=chronic obstructive pulmonary disease

Supplementary Figure 5: Forest plot of the comparison RDV vs SOT in subsets


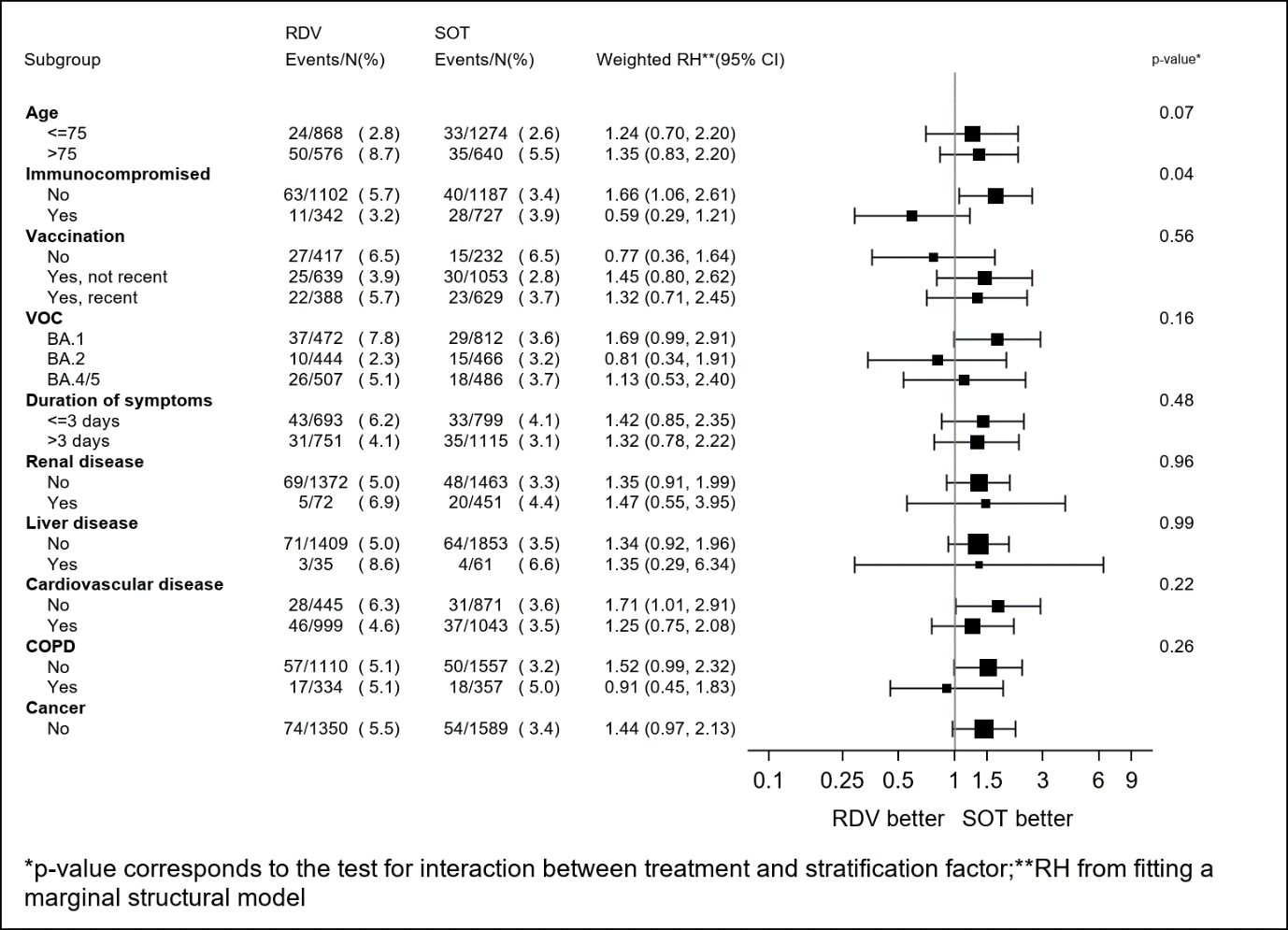


VOC=Variant of concern; COPD=chronic obstructive pulmonary disease

Supplementary Figure 6: Forest plot of the comparison RDV vs TIX/CIL in subsets


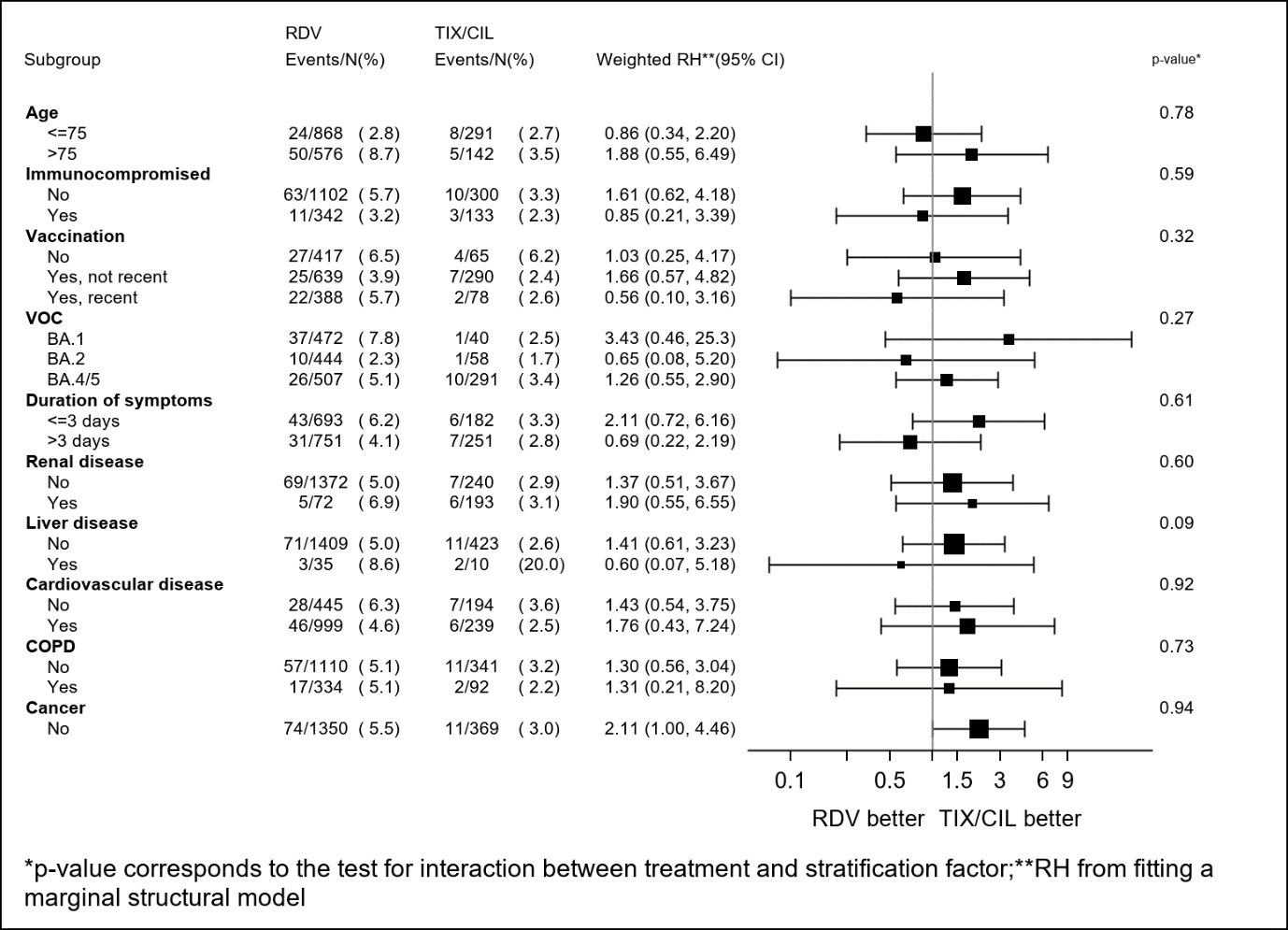


VOC=Variant of concern; COPD=chronic obstructive pulmonary disease

Supplementary Figure 7: Forest plot of the comparison SOT vs TIX/CIL in subsets


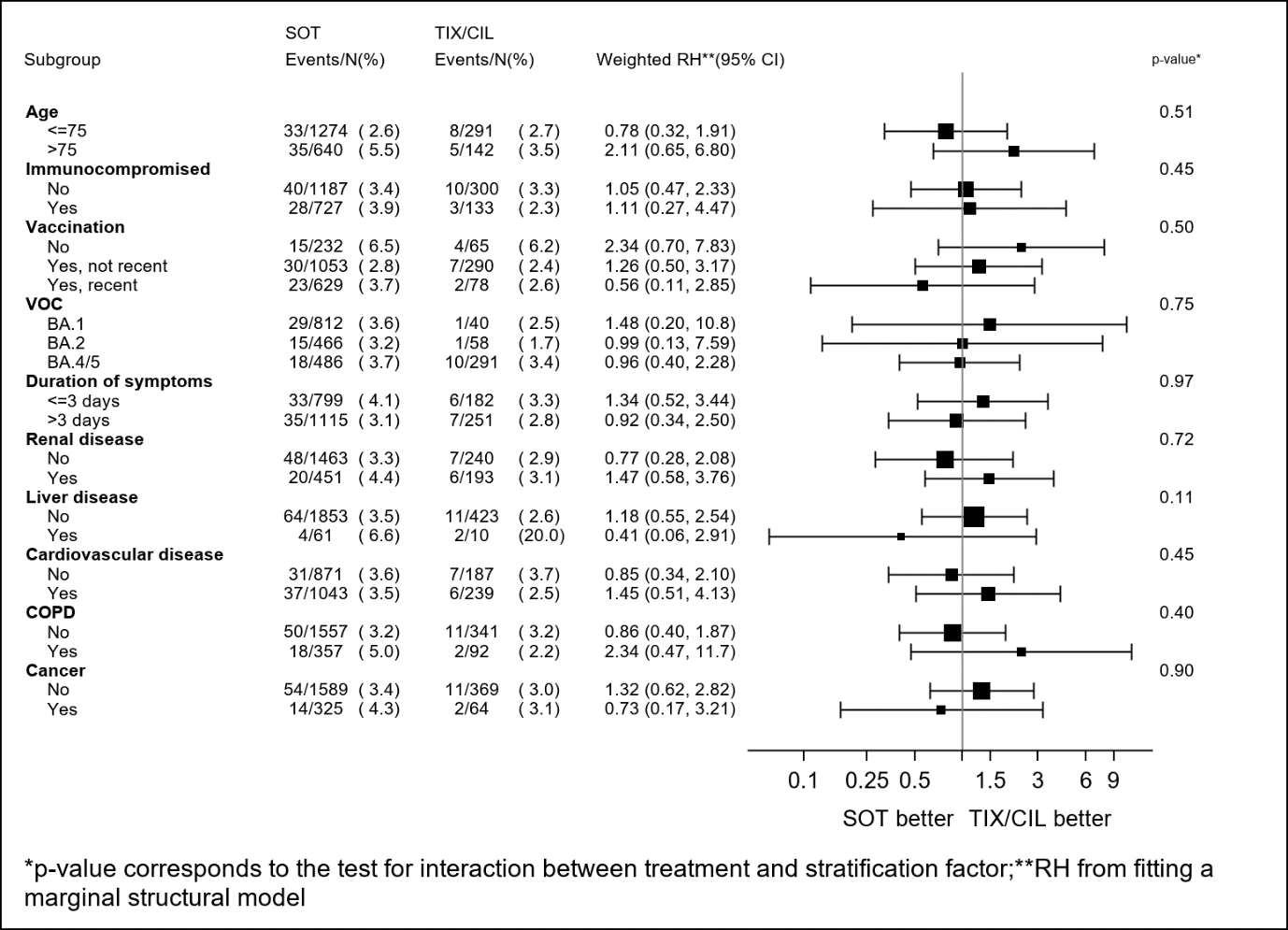


VOC=Variant of concern; COPD=chronic obstructive pulmonary disease
