# Supplementary material for: Comparative Analysis of Early COVID‐19 Treatment Efficacy in a Multicentric Regional Cohort in Italy: Emulation of a Series of Target Trials
Source: J Med Virol. 2025 May 6;97(5):e70379. doi: 10.1002/jmv.70379 (PMC12054396; doi:10.1002/jmv.70379)
Supplement: Supplementary file 2 — 5 Emulation table JMV final. [file JMV-97-e70379-s001.docx]

**Emulation table – Target Populations**

**General inclusion criteria for all trials**

**Definition of mild-moderate symptoms**

Mild-moderate symptoms were scores 1-5 of the WHO progression scale^28^ and defined according to the clinical spectrum classification of the NIH COVID-19 treatment guidelines^25^.

**AIFA definition of high-risk for progression**

Age >65 years, body mass index (BMI) >30, a previous diagnosis of diabetes mellitus, cardio- or cerebrovascular disease, cancer, renal or hepatic impairment, neurologic disease and immunocompromised status defined as primary or secondary immunodeficiency and/or ongoing treatment with immunosuppressive agents.

**General exclusion criteria for all trials:**

Pregnancy

Specific contraindications for the drugs available.

**Date of introduction of specific treatments in the outpatient setting**

TIX/CIL August 2022

NMV/r February 2022

RDV January 2022

MLP January 2022

SOT November 2021

**Additional inclusion/exclusion criteria for specific trials**

| **Trial** | **Additional inclusion criteria** | **Additional exclusion criteria** |
| --- | --- | --- |
| NMV/r vs. MLP | Duration of symptoms ≤5 days  Enrolment after Feb 2022 | Severe Renal impairment^1^ Severe hepatic impairment^2^ |
| NMV/r vs. RDV | Duration of symptoms ≤5 days  Enrolment after Feb 2022 | Severe Renal impairment^1^ Severe hepatic impairment^2^ |
| NMV/r vs. SOT | Duration of symptoms ≤5 days  Enrolment after Feb 2022 | Severe Renal impairment^1^ Severe hepatic impairment^2^ |
| NMV/r vs. TIX/CIL | Duration of symptoms ≤5 days  Enrolment after Aug 2022 | Severe Renal impairment^1^ Severe hepatic impairment^2^ |
| MLP vs. RDV | Duration of symptoms ≤5 days | Severe Renal impairment^1^ Severe hepatic impairment^2^ |
| MLP vs. SOT | Duration of symptoms ≤5 days |  |
| MLP vs. TIX/CIL | Duration of symptoms ≤5 days  Enrolment after Aug 2022 |  |
| RDV vs. SOT | None | Severe Renal impairment^1^ Severe hepatic impairment^2^ |
| RDV vs. TIX/CIL | Enrolment after Aug 2022 | Severe Renal impairment^1^ Severe hepatic impairment^2^ |
| SOT vs. TIX/CIL | Enrolment after Aug 2022 |  |

^1^ (eGFR<30) or dialysis

^2^ CHILD-Pugh C

Data on transplantation and use of anticoagulants were only available for a small subset of the cohort, so we could not apply further exclusions to the target population of trials including the NMV/r arm.
