# Supplementary material for: Comparative Analysis of Early COVID‐19 Treatment Efficacy in a Multicentric Regional Cohort in Italy: Emulation of a Series of Target Trials
Source: J Med Virol. 2025 May 6;97(5):e70379. doi: 10.1002/jmv.70379 (PMC12054396; doi:10.1002/jmv.70379)
Supplement: Supplementary file 3 — 6 Technical documentation JMV revision. [file JMV-97-e70379-s003.docx]

**Statistical Methodology**

We constructed stabilized weights by fitting a propensity score model for treatment, which included the following time-fixed factors measured at baseline as a linear predictor: age, history of vaccination, immunocompromised status (because of known high-risk in older, unvaccinated and immunocompromised^[[1]](#endnote-1)^) hepatic and renal disease (because predictors of outcome which also determine the choice of treatment), duration of symptoms (because indications for the time from symptoms onset to treatment initiation slightly varied by drugs) and calendar time of baseline (because of the change in the availability of drugs and the circulating SARS-CoV-2 sub-lineages over time). Censoring weights were constructed using the same model used to model treatment with the addition of time, which was modelled using restricted cubic splines with 3 knots at 1, 15, and 30 days. In a sensitivity analysis, we also included four binary time-fixed indicator variables for the participating sites in the propensity score model for both the treatment and censoring weights.

Vaccination status was defined as none or incomplete (less than three doses), full but not recent (>3 doses with the last dose administered >120 days before), and full recent (>3 doses with the last dose administered ≤120 days before). We performed a number of visual checks for the model assumptions by means of box plots of the stabilized weights, propensity score overlaps plots and standardized mean difference plots (Supplementary technical material). Because hazard ratios are not collapsible and do not work very well in the context of causal inference for non-rare events, we also used the risk difference as a causal estimand by means of double robust estimation with augmented inverse probability weighting (AIWP).

**Propensity score model**

Z=treatment

X=participants’ baseline characteristics

Pr(Z=1/ X=x) = logit p(z/x)= β_0_+ β_1_*age+ β_2_*vaccination+ β_3_*immunosuppression+β_4_*hepatic disease+β_5_*renal disease+β_6_*duration of symptoms+β_7_*calendar time

**Propensity scores overlap plots**

The graphs display the estimated density of the predicted probabilities that a participant treated with drug A could be allocated to drug B (blue colour) and the estimated density of the predicted probabilities that a participant treated with drug B could be allocated to drug A (red colour). Regardless of the trial, none of the plots indicates large probability mass near 0 or 1, and the two estimated densities have most of their respective masses in regions in which they overlap each other. Thus, there is no evidence that the positivity assumption is violated.

**Standardised mean difference plots**

These plots were done as a visual check to ensure that the weights could remove imbalance between arms. Standardized differences for each identified potential confounding factor are calculated as the weighted mean (or proportion) difference between groups divided by the weighted pooled standard deviation. A variable with a standardised difference below 10% (shown as the negligeable difference region in grey) is usually considered balanced. Most imbalances were small, and all could be corrected by the weights (differences labelled with an “+”). None of these plots suggested a misspecification of the weight models.

**NMV/r vs. MLP**

**NMV/r vs. RDV**

**NMV/r vs. SOT**

**NMV/r vs. TIX/CIL**

**MLP vs. RDV**

**MLP vs. SOT**

**MLP vs. TIX/CIL**

**RDV vs. SOT**

**RDV vs. TIX/CIL**

**SOT vs. TIX/CIL**

1. Mazzotta V, Cozzi Lepri A, Del Borgo C et al. Predictors of failure to COVID-19 early therapies and drugs efficacy comparison by emulation trial. Conference on Retroviruses and Opportunistic Infections (CROI) 2024; Denver, March 3-6, 2024. Poster Number #662 [↑](#endnote-ref-1)
